# RNA Expression Profiling of Human iPSC-Derived Cardiomyocytes in a Cardiac Hypertrophy Model

**DOI:** 10.1371/journal.pone.0108051

**Published:** 2014-09-25

**Authors:** Praful Aggarwal, Amy Turner, Andrea Matter, Steven J. Kattman, Alexander Stoddard, Rachel Lorier, Bradley J. Swanson, Donna K. Arnett, Ulrich Broeckel

**Affiliations:** 1 Department of Pediatrics, Children’s Research Institute, and Human and Molecular Genetics Center, Medical College of Wisconsin, Milwaukee, Wisconsin, United States of America; 2 Cellular Dynamics International Inc., Madison, Wisconsin, United States of America; 3 Department of Epidemiology, University of Alabama at Birmingham, Birmingham, Alabama, United States of America; Tokai University, Japan

## Abstract

Cardiac hypertrophy is an independent risk factor for cardiovascular disease and heart failure. There is increasing evidence that microRNAs (miRNAs) play an important role in the regulation of messenger RNA (mRNA) and the pathogenesis of various cardiovascular diseases. However, the ability to comprehensively study cardiac hypertrophy on a gene regulatory level is impacted by the limited availability of human cardiomyocytes. Human induced pluripotent stem cell-derived cardiomyocytes (hiPSC-CMs) offer the opportunity for disease modeling. Here we utilize a previously established *in* *vitro* model of cardiac hypertrophy to interrogate the regulatory mechanism associated with the cardiac disease process. We perform miRNA sequencing and mRNA expression analysis on endothelin 1 (ET-1) stimulated hiPSC-CMs to describe associated RNA expression profiles. MicroRNA sequencing revealed over 250 known and 34 predicted novel miRNAs to be differentially expressed between ET-1 stimulated and unstimulated control hiPSC-CMs. Messenger RNA expression analysis identified 731 probe sets with significant differential expression. Computational target prediction on significant differentially expressed miRNAs and mRNAs identified nearly 2000 target pairs. A principal component analysis approach comparing the *in* *vitro* data with human myocardial biopsies detected overlapping expression changes between the *in* *vitro* samples and myocardial biopsies with Left Ventricular Hypertrophy. These results provide further insights into the complex RNA regulatory mechanism associated with cardiac hypertrophy.

## Introduction

Left ventricular hypertrophy (LVH) is an adaptive response by the human heart to pressure and volume overload associated with hypertension, obesity and diabetes. It is one of the most potent risk factors for cardiovascular disease (CVD), including ischemic heart disease, chronic heart failure and CVD morbidity [1], [2]. Reversal of LVH has been shown to reduce the rate of cardiac events and stroke, independent of blood pressure levels [3]. The development of LVH is a complex process, with the enlargement of cardiomyocytes leading to an increase in the left ventricular mass (LV mass). This is accompanied by altered cell metabolism, signaling pathway changes and functional ventricular impairment [4], [5]. On a molecular level, cardiomyocyte hypertrophy is accompanied by re-activation of embryonic gene expression patterns and increased synthesis of contractile proteins [6].

Functional studies of LVH have primarily been conducted in animal models due to the limited availability of human cardiac tissue. The recent advances in stem cell technology, particularly induced pluripotent stem cells (iPSCs), offers the opportunity to generate and directly analyze human cardiomyocytes in healthy and disease states [7]. Ventricular cardiomyocytes generated from iPSCs recapitulate functional aspects of endogenous human cardiomyocytes and allow for more robust *in* *vitro* disease models in human cells for conditions such as LVH [8], [9]. To study the hypertrophic response in human cardiomyocytes, human induced pluripotent stem cell-derived cardiomyocytes (hiPSC-CM) were stimulated with endothelin 1 (ET-1). Endothelin 1 stimulation has been shown to cause neurohormonal stimulation of G-protein-coupled receptors resulting in modulation of phospholipase C and activation of cAMP pathways that cause biochemical and structural remodeling, which contributes to the development of hypertrophy [10]. In a recent study, Carlson et al demonstrate the use of an ET-1 stimulated hiPSC-CM assay to study cardiac hypertrophy *in* *vitro* [11].

MicroRNAs (miRNAs) have emerged as important regulators in cardiac development and regulation of RNA expression [12]. These RNAs are ∼22 nucleotide long, evolutionarily conserved, non-coding RNAs that post-transcriptionally regulate gene expression in a sequence-specific manner. There are currently over 1,500 known miRNAs in the human genome and each miRNA is expected to negatively control the regulation and stability of multiple messenger RNA (mRNA) targets. Several studies have investigated expression levels and identified miRNAs that modulate the key components involved in cardiac hypertrophy in animal models [13]–[16]. Here we investigate the response of hiPSC-CMs to ET-1 stimulation by expression profiling mRNA and miRNA. To identify RNAs in human cardiomyocytes that show differential expression upon induction of hypertrophy we performed a comprehensive expression analysis of both miRNAs and mRNAs in hiPSC-CMs and identified those that may be involved in the regulation of cardiac hypertrophy.

## Materials and Methods

### Cardiac hypertrophy model

**Figure 1:**
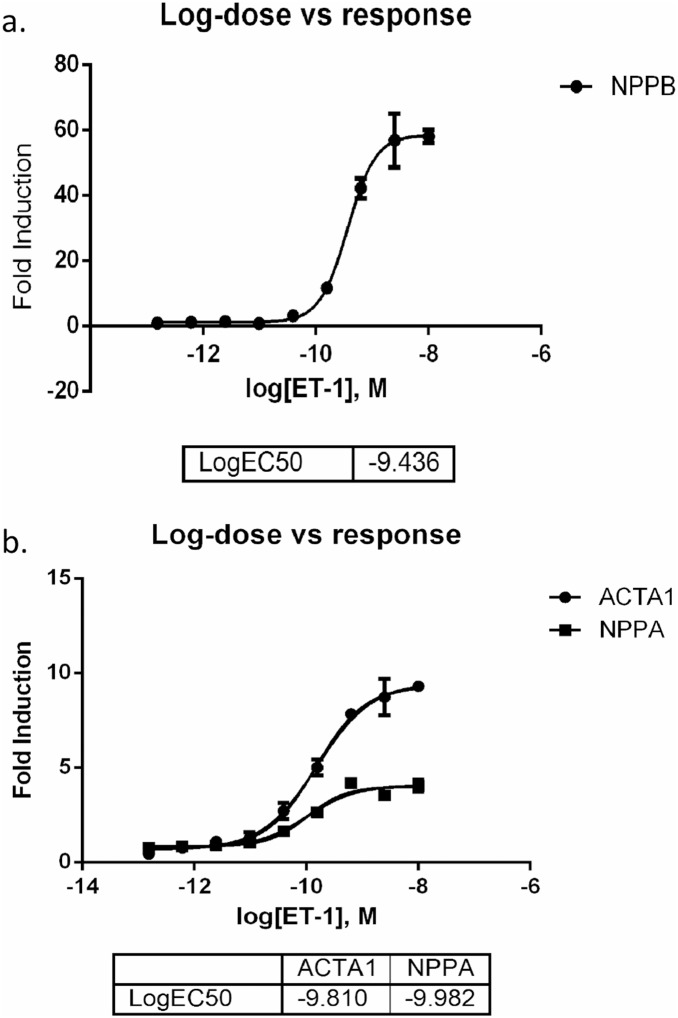
Dose vs. response plot for hypertrophy markers. RT-qPCR was used to measure the expression levels of (a) NPPB and (b) ACTA1 and NPPA as different ET-1 dose.

We utilized iCell Cardiomyocytes (iCell-CMs) derived from human iPSCs to characterize miRNA and mRNA expression patterns (Cellular Dynamics International, CDI). These cardiomyocytes are a highly pure ventricular population established as having functional properties similar to adult human cardiomyocytes [8]. All iCell-CMs were obtained from one single batch of cells. iCell Cardiomyocytes were plated at 2.0×10^4^ cells/well in a 96-well plate precoated with 0.1% gelatin solution. Several dose response and time course experiments were performed on these cells to determine the optimal ET-1 dosage, recovery and stimulation time that would give the maximum hypertrophic response. Figure 1a represents a dose vs. response plot for *NPPB* expression using relative quantification (RT-qPCR). After two days of recovery, the cells were cultured in William’s E medium supplemented with Cocktail B (1∶25) from the Hepatocyte Maintenance Supplement Pack (Life Technologies). After an additional 11 days of recovery, cells were stimulated for 18 h with ET-1 (Sigma Aldrich) at 10^−8^ M as recommended by the manufacturer. Experiments were performed in triplicate for both unstimulated controls (control-CM) and ET-1 stimulated cells (ET1-CM).

### RNA isolation

After 18 h, cells were harvested with Total RNA Purification 96-Well Kit (Norgen Biotek Corp.). Total RNA was extracted per manufacturer’s recommendations, resuspended in nuclease-free water, and quantified by UV spectrophotometry (NanoDrop 2000, Thermo Scientific). Quality of total RNA was evaluated using total RNA Pico chip analysis on Agilent 2100 Bioanalyzer. The total RNA analysis showed high quality RNA from which six cDNA libraries for RNA expression array and sequence capture were prepared.

### mRNA expression arrays

Messenger RNA expression was analyzed using GeneChip 3′IVT express arrays (Affymetrix). Fifty nanograms of total RNA for each of the samples, control-CMs and ET1-CMs, were prepared and analyzed on expression arrays following manufacturer’s instructions. Validation of mRNA expression levels was performed using TaqMan Gene Expression Assays (Life Technologies) on three classical hypertrophy markers, *NPPB, NPPA* and *ACTA1*. Relative quantification was performed per manufacturer’s recommendations on ABI 7900HT. Data analysis was performed using the delta-delta CT method, as part of the SDS 2.3 software package.

### miRNA sequencing

MicroRNA libraries from ribosomal RNA (rRNA) depleted total RNA (Ribo-Zero rRNA removal Kit, Epicentre) were prepared using Ion Torrent Total RNA Seq small RNA kit as per manufacturer’s recommendations (Life Technologies). Single-end sequencing was performed using the Ion Torrent Personal Genome Machine (PGM) sequencing platform. Following the Ion Sequencing Kit User Guide each sample was prepared, loaded and sequenced using small RNA library sequencing conditions on an Ion 318 chip. Validation of miRNA sequencing based expression levels on five known and five predicted novel mature miRNAs was done using RT-qPCR. TaqMan Small RNA Assays (Life Technologies) were performed on each miRNA in triplicate following manufacturer’s instructions. The data analysis was performed using the delta-delta CT method on ABI 7900HT using SDS 2.3 software.

### Data Analysis

SHRiMP2, a short read gapped aligner was used to map the raw sequencing reads against the human reference sequence (hg19) and a custom reference sequence set (containing miRBase v.20 known human miRNA hairpins, tRNA, rRNA, adapter and predicted novel miRNA hairpin sequences) [17]–[22]. Alignments with less than 17 bp matches and a custom 3′ end phred q-score threshold of 17 were filtered out. Subsequent analysis was conducted using only reads that represented known and predicted novel mature miRNAs. The miRDeep2 package (default parameters) was used to predict novel (as yet undescribed) miRNAs [23]. The consensus precursor sequences of the predicted novel miRNAs, which showed high sequence similarity with either snoRNA, rRNA or tRNA (using the Rfam database) were removed from further analysis [24].

Differential expression analysis of mRNA microarray and miRNA-Seq data was performed using different R/Bioconductor packages [25]. The affyQCReport package was used to perform QC on the microarrays [26]. The limma and affy software packages were used to process the microarray data [27], [28]. The mRNA data was combined with a public human myocardial biopsy dataset to study the overlapping expression profile of our *in* *vitro* model with *in* *vivo* myocardial biopsies. Myocardial left ventricular biopsies from male patients (n = 6) with isolated aortic stenosis and pronounced left ventricular hypertrophy undergoing aortic valve replacement were harvested either from hearts with normal ejection fraction (EF, >50%) or with low EF (<30%). Biopsies were further obtained from non-hypertrophied hearts with normal EF (>60%) from coronary artery disease patients undergoing coronary artery bypass graft surgery (n = 3). Total RNA isolated from biopsies was analyzed using Affymetrix HG-U133A and U133B GeneChip sets. To merge the two datasets, we first reduced the probeset data to gene level data and included only genes that were commonly differentially expressed between the two datasets. The prcomp method in R was used to perform the principal component analysis (PCA) on the merged datasets. The public dataset used for PCA is available in the ArrayExpress database (www.ebi.ac.uk/arrayexpress) under accession number E-MEXP-2296.

Gene set analysis to detect enriched disease phenotypes associated with the differentially expressed genes was performed using the WEB-based GEne Set AnaLysis Toolkit (WebGestalt) [29], [30]. WebGestalt uses a hypergeometric test for enrichment evaluation analysis. We set the multiple test adjustment parameter to be Benjamini and Hochberg method with an adjusted p-value less than or equal to 0.001 with a minimum of 10 genes in each disease category. We selected the human genome option as the reference set for enrichment analysis.

To study the miRNA expression profile of the hiPSC-CMs, we first quantified the miRNA-Seq data into known human and predicted novel mature miRNAs by using HTSeq v0.5.3p3 (http://www-huber.embl.de/users/anders/HTSeq). DESeq (v 1.12.1) was used to study the known and predicted novel miRNA expression differences between control-CMs and ET1-CMs [31]. In this study, differentially expressed genes that had a false discovery rate cutoff at 10% (FDR< = 0.1), a log_2_ fold change greater than 1.5 and less than −1.5 were considered significant. Significance filter for the known miRNAs included an FDR< = 0.1, a minimum up-regulation or down-regulation of 1.5 fold in their expression levels. For the predicted novel miRNAs, the FDR cutoff was changed to 20% or less.

Target gene prediction was performed using the TargetScan (version 6.2) database [32], [33]. This database uses a set of widely accepted primary target prediction criteria including pairing between the 5′ seed of the miRNA to a complementary 3′ UTR site of the mRNA, conservation of the miRNA binding site and free energy calculation based on the miRNA and mRNA UTR interaction. For a more detailed analysis of miRNAs and their target mRNAs, we restricted the analysis to the significant differentially expressed miRNAs and mRNAs. We also used miRTarBase (version 4.3), to identify targets that have been experimentally validated [34]. Targets for the predicted novel miRNAs were identified using psRNATarget, a small RNA target analysis server which is capable of accepting miRNA sequences as input to detect their potential binding sites [35]. This tool uses common target prediction criteria, including reverse complementary matching between miRNAs and target mRNA transcripts.

## Results

### Cardiac Hypertrophy Model

To characterize our *in* *vitro* model of cardiac hypertrophy, we performed several experiments including RT-qPCR on classical cardiac hypertrophy markers, global gene expression comparison with a human myocardial biopsy dataset and gene set enrichment analysis to detect enriched disease phenotypes.

**Figure 2:**
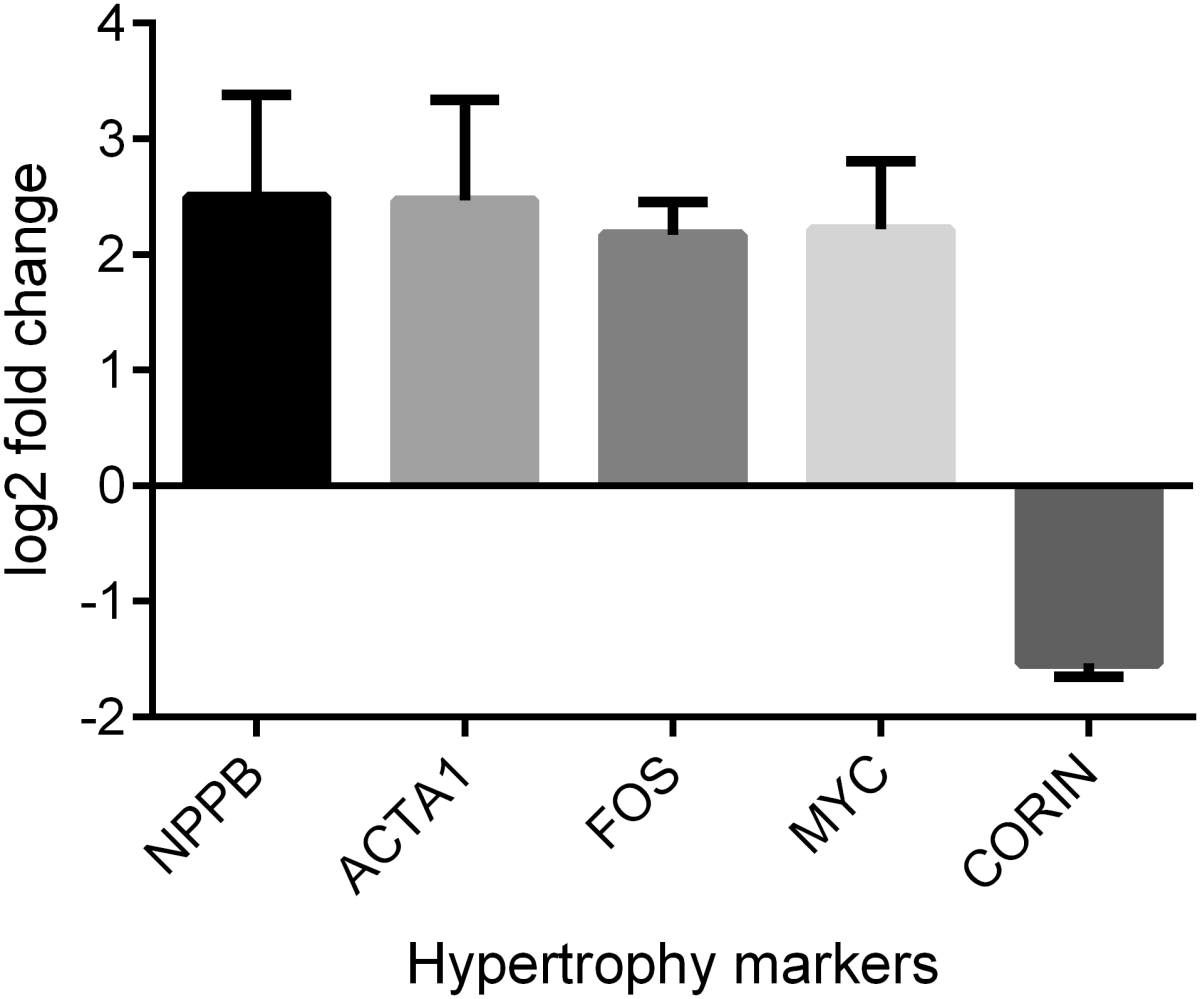
Cardiac hypertrophy marker expression. Bar plot showing the expression levels for some of the canonical hypertrophy markers in ET1-CM when compared with control-CM. The y-axis represents the mean + SD log2 fold change values taken from triplicate control-CM and ET1-CM experiments.

**Figure 3:**
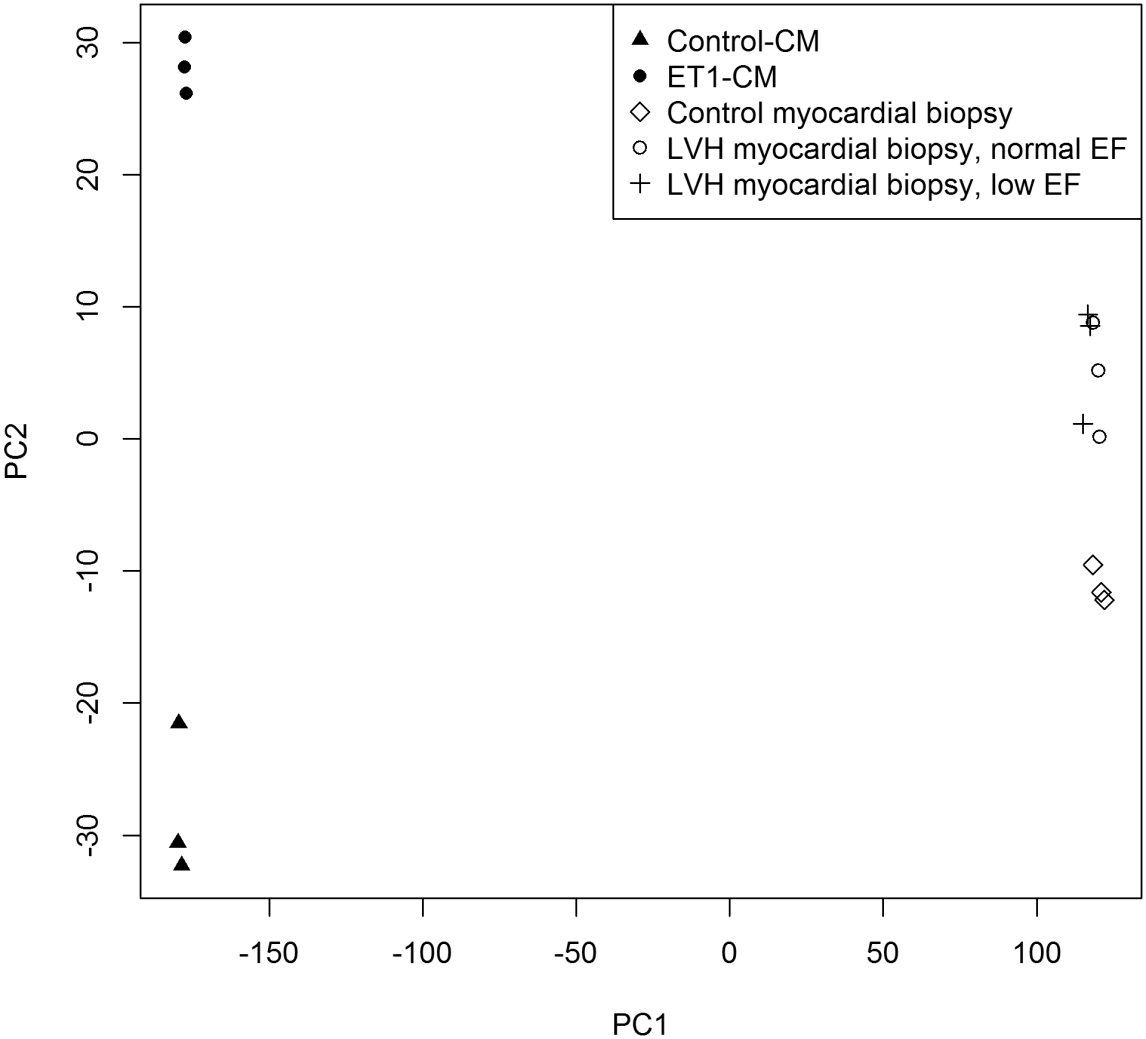
PCA plot comparing *in* *vitro* hiPSC model with *in* *vivo* human myocardial biopsies. Plot of first and second principal components (PC1 and PC2) for microarray expression data comparing ET-1 treated hiPSC-CMs with human myocardial biopsy with and without LVH data.

Firstly, we tested the expression levels of several classical cardiac hypertrophy markers including *NPPB, NPPA* and *ACTA1.* As expected in a hypertrophic phenotype, we observed increased expression levels for all these genes after ET-1 stimulation (Figure 1a and Figure 1b). Next, we ran mRNA expression arrays on these cells to analyze their global gene expression profiles associated with a hypertrophic response. The analysis of the mRNA expression arrays comparing ET1-CMs with control-CMs identified 731 out of 54,765 probe sets with significant differential expression (Table S1). These probe sets included many classical hypertrophy markers such as *NPPB, ACTA1* and *FOS* (Figure 2). These results were then evaluated against a publicly available dataset of human myocardial biopsies with and without LVH. The comparison of mRNA expression levels between our dataset and the biopsy dataset using the unsupervised PCA approach detected two dominant principal components (data not shown). Principal component 1 (PC1) represents the global differences between cell culture and biopsy data and principal component 2 (PC2) separates hypertrophic from normal samples in both the hiPSC-CM as well as the myocardial biopsy data (Figure 3). The genes that contribute to PC2 and show differential expression between control-CMs and ET1-CMs, characterize an overlapping hypertrophic response (Table S2). These include genes which are well established hypetrophy markers as well as genes like *MCM6* and *THBS1,* which have not been previously associated with hypertrophy. Finally, gene set analysis on these overlapping genes identified stress, hypertrophy and cardiovascular disease as highly enriched diseases associated with these genes (Table S3).

### miRNA-Seq analysis

Following reference alignment and read filtering of the miRNA-Seq data, between 863,000 and 1.9 million reads aligned to the custom reference set. Of the total mapped reads, 67% in the control-CMs and 85% in the ET1-CMs represented either known or predicted novel miRNAs. The remaining reads represented ribosomal (control-CMs vs.ET1-CMs: 6% vs. 3%), as well as transfer RNA (control-CMs vs. ET1-CMs: 26% vs.12%).

**Figure 4:**
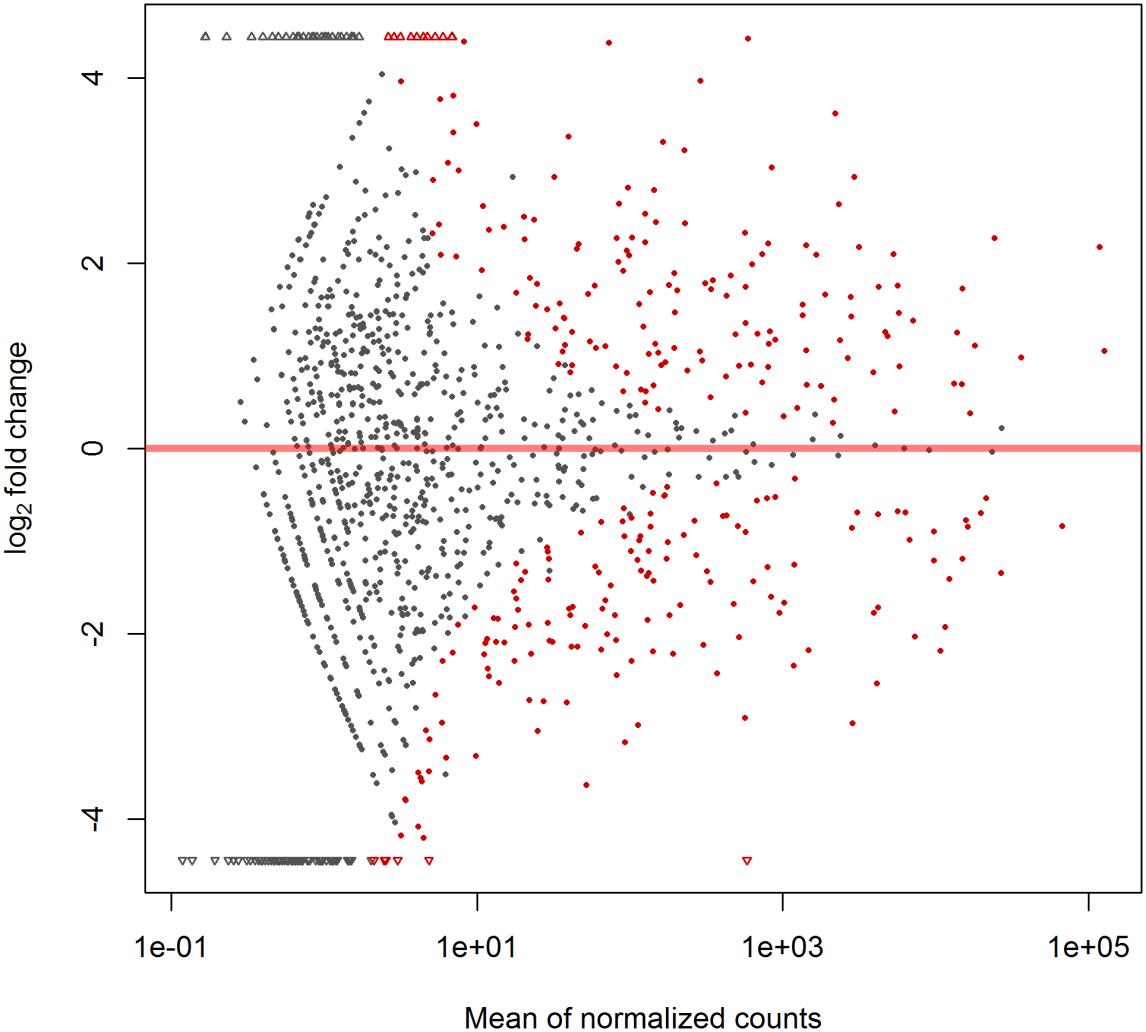
Differentially expressed known human miRNAs. MA-plot showing the differentially expressed known human mature miRNAs between control-CMs and ET1-CMs. The red solid dots represent the significant differentially expressed (FDR< = 0.1 and 1.5 fold change) known human mature miRNAs. See also Table S4.

We detected 836 known human mature miRNAs in the control-CMs and 769 in the ET1-CMs. Several mature miRNAs including hsa-miR-1-2, hsa-miR-23a-3p and hsa-miR-145-5p were highly abundant in all sequenced samples. We also observe miRNAs with moderate abundance, hsa-mir-22-3p, hsa-mir-208a-3p in addition to low abundant transcripts like hsa-mir-195. Expression analysis found 292 known mature human miRNAs to be differentially expressed (Figure 4 and Table S4).

Based on our miRNA-Seq data, we predicted 506 sequences to be potentially novel, as yet undescribed miRNAs. Of these, 34 predicted mature miRNAs had significant differential expression (Table S5). Providing further evidence for the prediction analysis, several of these novel miRNAs shared a common seed with known miRNAs from other species like mouse, mmu-miR-707 and mmu-mir-21b.

### miRNA RT-qPCR validation

**Figure 5:**
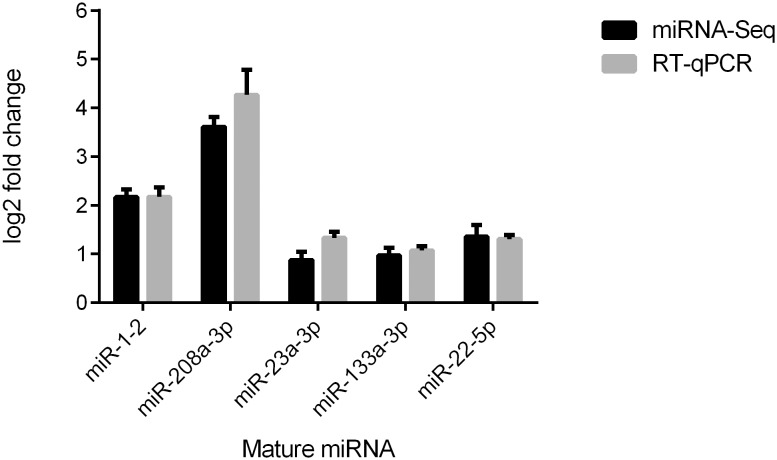
Known mature miRNA validation with RT-qPCR. Comparison of RT-qpCR and miRNA-Seq derived log_2_ fold change for a subset of known human mature miRNAs between control-CMs and ET1-CMs. The mean values taken from triplicate experiments are plotted with standard deviation error bars.

In order to validate the expression profiles of the miRNAs detected, we performed RT-qPCR on a subset of five known human mature and five of our predicted novel miRNAs. As shown in Figure 5 and Figure S1, we observed similar expression profiles from both RT-qPCR and miRNA-Seq for all the selected known and predicted novel miRNAs between control-CM and ET1-CM samples.

### Identification of putative miRNA gene targets

We used the TargetScan target prediction database to detect the potential miRNA-mRNA target pairs. After filtering target pairs to include only significantly differentially expressed genes and miRNAs, we obtained a total of 1,922 predicted miRNA-mRNA pairs represented by 309 genes and 174 known mature human miRNAs. Based on known functional effects of miRNAs as inhibitors of mRNA, we divided the miRNA-mRNA pairs into two groups: (a) up-regulated mRNA and down-regulated miRNA, and (b) down-regulated mRNA and up-regulated miRNA. We identified 157 significantly up-regulated mRNAs predicted to be primary targets of the observed significantly down-regulated known mature human miRNAs (Table S6) and 152 down-regulated genes that are predicted primary targets of the up-regulated miRNAs (Table S7).

Subsequently, we also used miRTarBase to detect the experimentally validated miRNA-mRNA target pairs. After filtering these pairs to only include targets with significant differential expression of the miRNAs and mRNAs, we identified 320 target pairs which represent a combination of 204 genes and 54 known mature human miRNAs. Selecting pairs based on an inverse relationship between miRNA and gene expression levels, we identified a total of 105 miRNA-mRNA pairs that show an increase in mRNA expression with a reduced expression of miRNAs upon ET-1 stimulation (Table S8). In addition, 215 target pairs showed a reduced mRNA expression while their respective miRNAs increased (Table S9). These include pairs that have already been established to regulate cardiac hypertrophy in other models and several pairs where either the miRNA or its corresponding target has not yet been implicated in hypertrophy. Target prediction analysis on the novel miRNAs found potential targets for nine of the predicted novel miRNAs (Table 1).

**Table 1:** Predicted novel miRNAs and their mRNA targets.

Novel miRNA ID	log_2_ Fold Change	False Discovery Rate (FDR)	Mature Sequence	Predicted Targets
chr5_21180	3.03	0.1043	uucuggaacauugggcucagu	GPM6B
				SLC9A7
				RALGPS1
				RD3
				RASGRF2
chr16_25512	Repressed in ET-1	0.1170	uguccacccucccccacuccaga	SLC4A1
				C1orf189
				CXXC4
				LAX1
				ATP8B2
chr13_36699	−1.93	0.1438	ucuucucagaggggcaccccacugu	CBX5
				CCDC129
chr2_7281	Repressed in ET-1	0.1438	ugggccucgcgcugaucagcag	CCDC74A
				CCDC74B
chr11_21008	Repressed in ET-1	0.1518	gcaggaagagacgcagcagcuugu	KIAA1671
				RIC3
				ZNF446
				TLCD2
				CBLN3
				FGF14
chr1_4434	−2.61	0.1518	gaacaacccuauccucuuccaga	ZNF619
				GOLGA5
chr5_23024	3.72	0.1543	cugccaucuggugccagccuuu	MEF2C
				WIZ
				LOC100506422
				ELP6
chr22_43617	−1.93	0.1543	ucccacugcuucacuugacuagc	CDK6
				RAB37
				SLC7A1
				KIAA0408
				SOGA3
chr13_35963	3.93	0.1625	uaugugccuaguggcugcugucu	LHCGR
				TPGS2

List of predicted novel miRNAs with significant differential expression and their predicted mRNA targets. The miRNAs with “Repressed in ET-1” log_2_ fold change have a normalized read count of zero in ET1-CMs. See also Table S2.

## Discussion

Recent developments in stem cell technology, particularly iPSCs, offer the opportunity to directly study human cardiomyocytes in healthy and disease states [9]. Prior studies conducted in animal models have investigated the role of miRNAs in the development of LVH and cardiac hypertrophy [36]–[42]. Growing evidence of transcriptional changes and regulation differences between various animal and human disease models highlight the need for comparative studies in human cardiac tissue [43], [44]. In this study, we recapitulate a specific aspect of the cardiac hypertrophy phenotype in hiPSC-CMs by stimulating them with ET-1, a potent vasoconstrictor and a well established modulator of cardiac hypertrophy in humans [45], [46]. Our analysis of hiPSC-CMs elucidates RNA expression and regulation in a human derived cell line which when combined with myocardial biopsy data may provide a more comprehensive picture of cardiac hypertrophy in humans. In this study, we induce cardiomyocyte hypertrophy using ET-1. The observed expression changes are specific to this inducer of hypertrophy. Further studies will be required to test the impact of other well-established pro-hypertrophic agents.

When using an *in* *vitro* system for disease modeling in humans it is critical to characterize and validate it to confirm it’s efficiency. To achieve this for our cardiac hypertrophy model, we first performed RT-qPCR on several classical hypertrophy markers to detect any changes in their expression levels between the control-CMs and ET1-CMs. We observed significant expression changes for several of the studied markers. However, just looking at specific genes may not be sufficient to validate a disease model. Thus, we ran mRNA expression arrays on these cells to study their global gene expression profiles. Evaluating these gene expression levels against human tissue samples, such as patient cardiac biopsy strengthens the confidence in a disease model. However, with the limited availability of tissue samples it can be challenging to perform such a validation. In this study, to alleviate this limitation we compared our mRNA expression dataset with a publicly available human myocardial left ventricular biopsy dataset that includes samples with and without LVH. To perform this analysis we used a principal component analysis approach. This is a unique methodology to compare these datasets as it is an unsupervised methodology and thus allows us to describe group expression changes driven only by the strength of their similarity and correlation. Principal component 1 clustered the correlated transcripts and separated the two experimental systems, hiPSC-CMs and myocardial biopsy. This separation was expected and represents the complexity of other endogenous factors, including the cardiac extracellular matrix, fibroblasts and other vascular tissues in the biopsy data. Once these system differences were accounted for, variance was driven by the differences in phenotype (hypertrophy vs. control). Interestingly, the most highly loaded gene on PC2 was the canonical hypertrophy marker *NPPB*. B-type natriuretic peptide (BNP) is a cardiac hormone produced by the ventricles during cardiac differentiation. It is re-expressed in response to increased cardiac stress leading to natriuresis, vasodilation and inhibition of the renin-angiotensin system. Re-expression of *NPPB*/BNP and its secretion into plasma has become an important clinical biomarker for diagnosis and treatment of heart failure and LVH [47]–[49]. We identified the genes that were loaded in PC2 and also show significant differential expression in our model.

A gene set enrichment analysis was then performed on the overlapping genes to identify the major disease phenotypes associated with these genes. From this analysis, we detected stress, cardiovascular disease and hypertrophy to be some of the significantly enriched diseases. These results provide strong evidence that the observed *in* *vitro* changes induced by ET-1 recapture a specific subset of expression changes observed *in* *vivo* in humans with LVH. This approach of comparing the relevant phenotype in the originating disease tissue and the hypertrophic hiPSC-CM provides a novel methodology for validation of disease models.

There has been increasing evidence on the role played by miRNAs in the regulation of cardiovascular development and disease mechanisms. A cardiac hypertrophy model based on iPSC-derived cardiomyocytes provides a unique system to study miRNA expression changes associated with the disease phenotype. To interrogate the hypertrophy driven miRNA expression differences in our hiPSC-CMs, we performed miRNA-Seq and expression analysis. We detected more than 250 known human mature miRNAs with significant differential expression between ET1-CMs and control-CMs. These include known miRNAs both associated with and those not previously linked to cardiac hypertrophy.

Of the previously established hypertrophy miRNAs, we found hsa-miR-23a-3p, hsa-miR-22-3p and hsa-miR-208a-3p to have significant differential expression. All these miRNAs were up-regulated in ET1-CMs, which is consistent with several studies focused on these markers in different animal models of cardiac hypertrophy [36], [38], [41], [42]. Interestingly, we detected hsa-miR-1-2 to be significantly up-regulated in this study. However, it’s expression has been shown to be attenuated in cardiac hypertrophy in rat models [39], [40]. This observation highlights the dynamic role of miRNA expression in the pathogenesis of disease under different experiemental conditions.

MicroRNA regulation of hypertophy may be caused by direct (primary) or indirect (secondary or tertiary) targeting of genes. To investigate these mechanisms of gene regulation, we combine our miRNA and mRNA expression data to detect potential miRNA-mRNA target pairs. These pairs include primary target genes that encode a wide variety of functional proteins. These include *MYC* and *FOS* which have been shown to play a key role in cell proliferation and transformation and are also involved in the regulation of cardiac hypertrophy [50], [51]. In addition, genes like *MAP3K9* and *MAP2K6* that are essential components of the mitogen-activated protein kinase signal transduction pathways have been linked to heart failure and cardiac hypetrophy are also detected [52]. Other significant predicted targets include transforming growth factors like *TGFB2* and transforming growth factor beta receptor, *TGFBR3*. The three different isoforms of TGF-beta have been shown to play a significant role in myocardial infarction and in the regulation of hypertrophic cardiac remodeling [53].

One major advantage of a miRNA sequencing approach lies in the ability to identify potential miRNAs that remain undetected and thus undescribed using other approaches. This is of particular importance since the annotation of human miRNAs is still limited. We predict several novel miRNAs from the sequencing data based on differential expression. Most of these predicted miRNAs had the characteristic miRNA stem loop structure and some also share a common seed with known mouse miRNAs, indicating possible sequence conservation across species.

Several of the predicted gene targets of our novel miRNAs were involved in the regulation of cardiac disease and hypertrophy. These targets include: *MEF2C*, a gene known to play a critical role in cardiovascular development and cardiac hypertrophy; *CDK6*, a member of the cyclin D signaling pathway the inhibition of which impairs cardiac hypertrophy in both *in* *vitro* and *in* *vivo*; and *TLCD2,* a gene identified in GWAS analysis as a strong candidate associated with LV mass [54]–[56]. Further investigation needs to be performed on these novel miRNAs to elucidate their potential role in the regulation of cardiac hypertrophy.

To summarize, left ventricular hypertrophy remains a major risk factor for cardiovascular disease. Due to the limitations in the availability of cardiac tissue and the documented differences between human and animal models, hiPSC-CMs provide a viable alternative to study cardiac disease in humans. However, the use of iPSC derived cells is significantly governed by the disease of interest. For example, in case of a complex phenotype like cardiac hypertrophy, besides mimicing the genetic aspect, it is imperative to regulate the extrinsic, non-genetic component of the disease process. Using a cardiac hypertrophy stimulant like ET-1 instead of patient specific cell lines gives us the opportunity to establish an unbiased model to focus on identifying the underlying molecular pathways that help regulate the disease mechanism. Once established, we can use this model to perform future studies to focus on patient specific regulation of the disease process. With current treatment options limited, miRNAs provide a new class of potential therapeutic targets. In this study, we provide the first comprehensive RNA expression dataset for a hiPSC-derived cardiomyocyte model of cardiac hypertrophy. While our method of recapitulating the disease phenotype using ET-1 captures only a subset of the complex cardiac hypertrophy disease mechanism, it provides significant insights into hypertrophic regulatory pathways. Utilizing hiPSC-CMs and comparing our expression results to both animal models and human biopsies allows us to take the next step in identifing novel miRNAs and gene targets that may play a role in human disease.

## Supporting Information

Figure S1 **Predicted novel miRNA expression validation with RT-qPCR.** Bar plot with expression levels obtained from RT-qPCR on a subset of the differentially expressed predicted novel miRNAs. The values are mean log2 fold change from triplicate ET1-CM and control-CM experiments with standard deviation error bars.
(TIF)

Table S1 **Differentially expressed mRNA.** This list includes mRNA probe sets with significant differential expression between the control-CM and ET1-CM libraries.
(XLSX)

Table S2 **Differentially expressed genes contributing to principal component 2 when comparing** ***in*** ***vitro*** **hiPSC-CMs with** ***in*** ***vivo*** **human myocardial biopsies.**
(XLSX)

Table S3 **Disease enrichment analysis on differentially expressed genes that overlap with the myocardial biopsy data.** The analysis used the whole genome for enrichment with a Benjamini-Hochberg corrected significance level of 0.001 or less and a minimum 10 genes in each disease.
(XLSX)

Table S4 **Differentially expressed known human mature miRNAs.** The list of the known mature miRNAs observed to have significant differential expression between control-CMs and ET1-CMs. Related to Figure 4.
(XLSX)

Table S5 **Differentially expressed predicted novel miRNAs.** This is the list of the predicted miRNAs with significant differential expression between control-CMs and ET1-CMs. The miRNAs with “Repressed in ET-1” log2 fold change have zero normalized read counts in ET1-CMs.
(XLSX)

Table S6 **Computationally predicted up-regulated mRNA targets of down-regulated miRNAs.** These miRNA-mRNA target pairs were detected using the TargetScan database. This list includes known human mature miRNAs that had reduced expression after ET-1 stimulation with their up-regulated mRNA targets.
(XLSX)

Table S7 **Computationally predicted down-regulated targets of up-regulated miRNAs.** This is a list of predicted miRNA-mRNA target pairs as detected by the TargetScan database. Up-regulated miRNAs with down-regulated predicted targets are included in this list.
(XLSX)

Table S8 **Experimentally validated up-regulated mRNA targets of down-regulated miRNAs.** These miRNA-mRNA target pairs were identified using the miRTarBase database of experimentally validated miRNA targets. This list includes known human mature miRNAs that had reduced expression after ET-1 stimulation and their mRNA targets were observed to be up-regulated in this study.
(XLSX)

Table S9 **Experimentally validated down-regulated mRNA targets of up-regulated miRNAs.** These miRNA-mRNA target pairs were identified using the miRTarBase database of experimentally validated miRNA targets. miRNAs that were up-regulated after ET-1 stimulation and their down-regulated targets are included in this list.
(XLSX)
