# Supplementary figures and images for: RNA Expression Profiling of Human iPSC-Derived Cardiomyocytes in a Cardiac Hypertrophy Model
Source: PLoS One. 2014 Sep 25;9(9):e108051. doi: 10.1371/journal.pone.0108051 (PMC4177883; doi:10.1371/journal.pone.0108051)

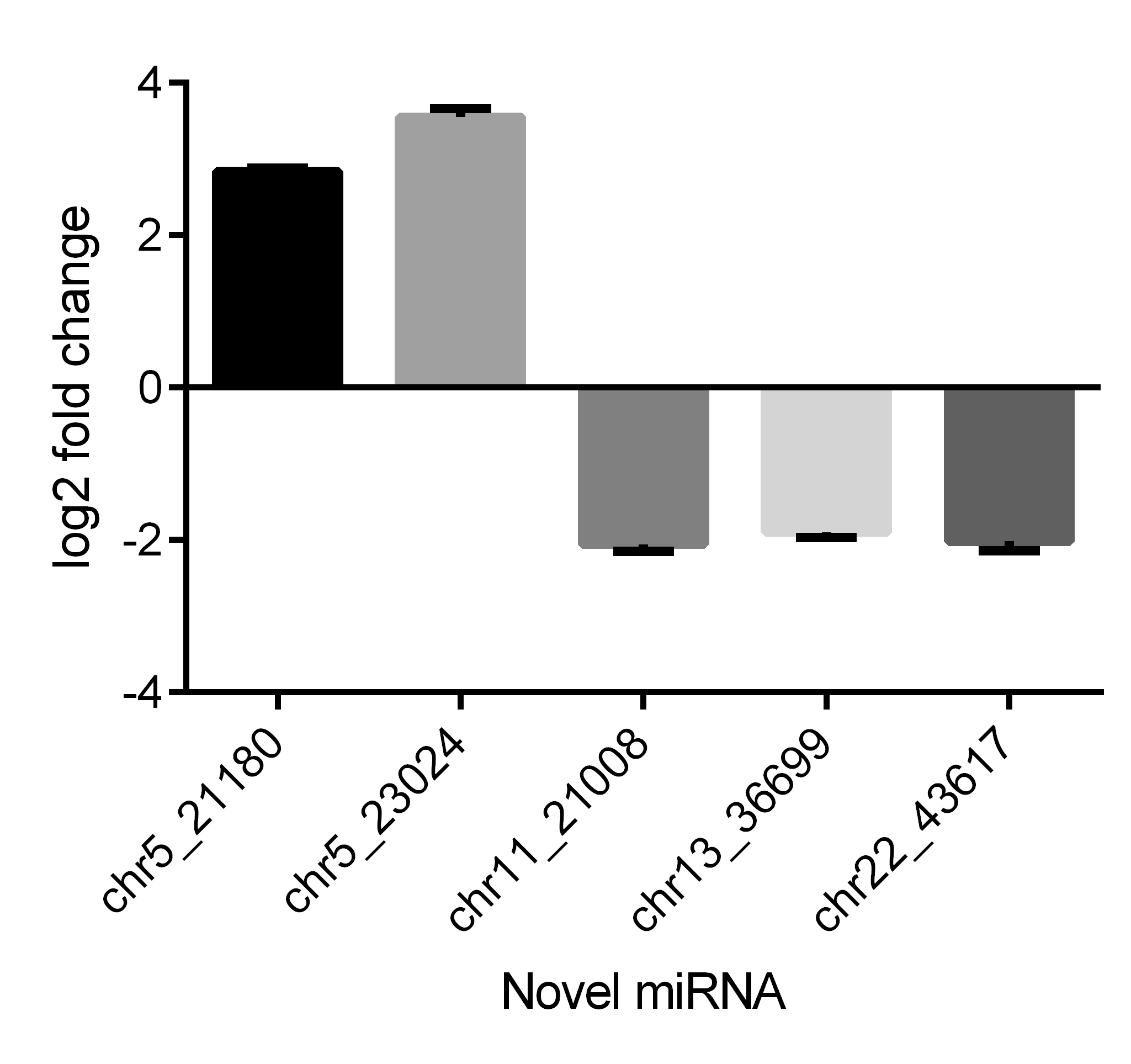

Supplement: Figure S1 — Predicted novel miRNA expression validation with RT-qPCR. Bar plot with expression levels obtained from RT-qPCR on a subset of the differentially expressed predicted novel miRNAs. The values are mean log2 fold change from triplicate ET1-CM and control-CM experiments with standard deviation error bars. (TIF) [file pone.0108051.s001.tif]
